# Using high-resolution LiDAR-derived canopy structure and topography to characterise hibernaculum locations of the hazel dormouse

**DOI:** 10.1007/s00442-023-05429-3

**Published:** 2023-08-06

**Authors:** Leonardo Gubert, Fiona Mathews, Robbie McDonald, Robert J. Wilson, Ruud P. B. Foppen, Pim Lemmers, Maurice La Haye, Jonathan Bennie

**Affiliations:** 1 Centre for Ecology and Conservation, University of Exeter, Penryn, TR10 9FE UK; 2 School of Life Sciences, University of Sussex, Brighton, BN1 9QG UK; 3 Environment and Sustainability Institute, University of Exeter, Penryn, TR10 9FE UK; 4 Department of Biogeography and Global Change, Museo Nacional de Ciencias Naturales (MNCN-CSIC), 28770 Madrid, Spain; 5 Department of Animal Ecology and Physiology, Radboud Institute for Biological and Environmental Sciences, Radboud University, P.O. Box 9100, 6500 GL Nijmegen, The Netherlands; 6 Natuurbalans-Limes Divergens, Toernooiveld 1, 6525 ED Nijmegen, The Netherlands; 7 The Dutch Mammal Society, Toernooiveld 1, 6525 ED Nijmegen, The Netherlands; 8 Centre for Geography and Environmental Science, University of Exeter, Penryn, TR10 9FE UK

**Keywords:** Hibernation, Remote sensing, *Muscardinus avellanarius*, Habitat selection, LiDAR

## Abstract

The hazel dormouse is predominantly an arboreal species that moves down to the ground to hibernate in the autumn in temperate parts of its distributional ranges at locations not yet well understood. The main objective of this study is to test whether environmental characteristics surrounding hazel dormouse hibernacula can be identified using high-resolution remote sensing and data collected in situ. To achieve this, remotely sensed variables, including canopy height and cover, topographic slope, sky view, solar radiation and cold air drainage, were modelled around 83 dormouse hibernacula in England (*n* = 62) and the Netherlands (*n* = 21), and environmental characteristics that may be favoured by pre-hibernating dormice were identified. Data on leaf litter depth, temperature, canopy cover and distance to the nearest tree were collected in situ and analysed at hibernaculum locations in England. The findings indicated that remotely sensed data were effective in identifying attributes surrounding the locations of dormouse hibernacula and when compared to in situ information, provided more conclusive results. This study suggests that remotely sensed topographic slope, canopy height and sky view have an influence on hazel dormice choosing suitable locations to hibernate; whilst in situ data suggested that average daily mean temperature at the hibernaculum may also have an effect. Remote sensing proved capable of identifying localised environmental characteristics in the wider landscape that may be important for hibernating dormice. This study proposes that this method can provide a novel progression from habitat modelling to conservation management for the hazel dormouse, as well as other species using habitats where topography and vegetation structure influence fine-resolution favourability.

## Introduction

Detailed knowledge of environmental conditions suitable for individual species across the landscape is crucial for understanding species’ ecology and conservation. The use of remotely sensed data to generate topographic data (Mallet and Bretar 2009; Passalacqua et al. 2015; Rose et al. 2015) and information on canopy structure (Dalagnol et al. 2021; Frolking et al. 2009; Liu et al. 2020) is a powerful tool that has increased knowledge of woodland species distributions (De Frenne et al. 2019; Stark and Fridley 2022).

As remote sensing technology constantly develops, increasingly high-resolution data have allowed researchers to investigate habitat quality and species distribution at increasingly fine scales (de Vries et al. 2021) as well as to improve models of species’ ecological niches, by including measures such as habitat quality, and seasonal or life-cycle events (Leitão and Santos 2019).

Airborne Light Detection and Ranging (LiDAR) is an established and reliable means of generating data on the physical structure of topography, vegetation and man-made structures that have been used for diverse purposes, including forestry (Næsset and Økland 2002), landscape mapping (Wang et al. 2021), habitat modelling and assessment (Getzin et al. 2021; Hagar et al. 2020), landcover types and habitat classification (Koma et al. 2021), natural resources management (Garabedian et al. 2017) and wildfire modelling (Botequim et al. 2019; Rosa and Stow 2014). Airborne LiDAR at resolutions of < 1 m is capable of providing information on the scale of individual trees (Jaskierniak et al. 2021; Lichstein et al. 2010), which can be important for a variety of forestry activities and for environmental modelling (Khosravipour et al. 2015). Such high-resolution spatial data also have the potential to identify landscape features that are important for the survival of individual organisms, whilst highlighting ecological variations at multiple scales, in local areas, regions and countries. Such approaches offer greater accuracy than many methods that are traditionally used in landscape ecology, for example, coarser-scale remote sensing and land cover mapping. The greater level of detail that can be extracted from LIDAR offers a novel advance for habitat modelling and/or species distribution modelling.

To improve the scope of ecological models it is important to take into consideration the spatial and temporal distribution of climate at the landscape scale. For habitat modelling, microclimate has long been acknowledged as a key factor in the spatial distribution of numerous species. Landscape-scale modelling of microclimates can be a powerful tool for conservation actions, including habitat creation, restoration, management, and species reintroductions (Bennie et al. 2010; de Vries et al. 2021; Lembrechts et al. 2019; Massimino et al. 2020).

Many species use hibernation as a strategy to minimise the amount of energy spent in thermoregulation and, for this purpose, a suitable hibernaculum location capable of providing appropriate conditions is crucial to increase survival chances. For some species, such as bats (De Bruyn et al. 2021), ground squirrels (Goldberg et al. 2020) and bears (Cisneros-Araujo et al. 2021), these requirements may be different from those during the active season (Goldberg et al. 2020) and can include factors such as stable temperatures, correct level of humidity, safety from predators, low susceptibility to flooding, proximity to important resources, and others.

The hazel dormouse *Muscardinus avellanarius* is a mainly nocturnal animal that spends most of its active season in the shrub layer or canopy and, in northern European countries, they move down to the ground to hibernate in the autumn where they stay until spring at locations not yet well understood (Juškaitis 2014). During the pre-hibernation period in temperate climates, the tendency for dormice to enter daily torpor increases with decreasing ambient temperature and day length (Mills 2012). By the beginning of November, dormouse use of natural nests above ground and artificial nest boxes tends to decrease as they prepare for hibernation at ground level (Juškaitis 2014). The preference for dormice to overwinter on the ground is thought to be associated with more stable temperatures at which to hibernate. A humid site is preferable to prevent the animal from dehydration over winter (Morris 2004), where they have always been found to hibernate alone in individual nests made from plant material occupying all the interior nest volume available (Gubert et al. 2022; Vogel and Frey 1995).

In Britain, pre-breeding population densities of hazel dormice range from 5 to 8 adults/ha (Bright and Morris 1996) but can be less, depending on quality and type of habitat. With their low population density, behaviour and nest size, dormouse hibernacula are notoriously difficult to find and apart from some targeted studies (Gubert et al. 2022; Lemmers 2022; Verbeylen et al. 2017; Vogel and Frey 1995; Walhovd and Jensen 1976), hibernation of wild animals remains one of the least known aspects of the species’ ecology.

The minimum area of continuous habitat able to support hazel dormouse populations in the long term varies hugely with habitat quality and connectivity (Bright et al. 2006). Adult hazel dormice are sedentary and have fixed home ranges of around 0.5–1.0 ha during the active season. However, there is little information on their preferred hibernaculum and when it comes to dormouse conservation, there is limited evidence of where they hibernate and whether there is more that can be done in terms of habitat management as well as improving associated protocols for such activities to increase the chances of over-winter survival.

Remote sensing has been successfully used for investigations in hazel dormouse spatial ecology, capable of identifying short-term habitat preferences (Goodwin et al. 2018) as well as establishing broad-scale habitat descriptors associated with the current distribution populations (Cartledge et al. 2021).

In this study, we have collected information on where hazel dormouse hibernation takes place in England and the Netherlands to identify habitat features at local and landscape scales, through an approach integrating high-resolution remote sensing and data collected in situ. We have also investigated the feasibility of a predictive model to aid the identification of potential hibernaculum locations to aid species’ conservation.

## Methods

### Study areas

**Fig. 1:**
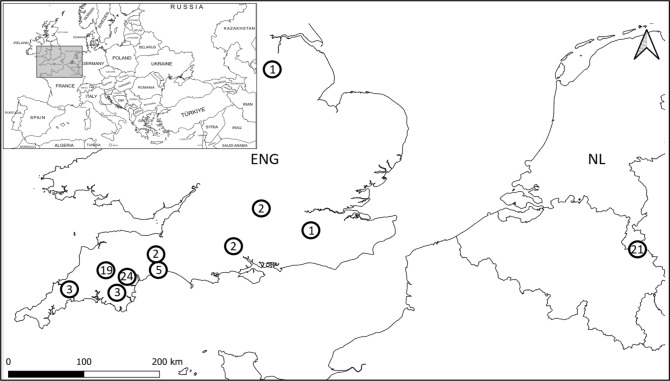
Location of the study areas in England and the Netherlands. Dots represent hibernaculum location and the numbers are hibernacula clusters recorded in the area

We analysed 12 study areas (Fig. 1) where dormouse hibernacula had been located by a range of methods, comprising broadleaved, coniferous, and mixed woodlands, roadside habitats, hedgerows, scrub, and gardens, in England and the southern Netherlands. These habitat types of variable age and structure are typical of those occupied by hazel dormice and often form a connected mosaic of habitats in the wider landscape. The study areas in these two countries are similar to a certain extent in their underlying geology, with the Netherlands comprising eroded limestone with high contents of loess and clay, and the sedimentary formations of mudstone, siltstone and sandstone in England, which is also subject to higher winter rainfall.

### Methods used to locate hibernacula

Hazel dormouse hibernacula were located through telemetry in both countries with additional records generated from systematic searches and incidental observations in England. Hibernation nests were identified by their typical structure and characteristic location at ground level.

#### Radio telemetry

Wild dormice were captured under licence from nest boxes and tubes installed at locations where the species’ presence had been confirmed. In England, dormouse captures were timed just before the arrival of cold weather fronts during autumn 2016, when the lower temperature was expected to trigger hibernation and while dormice were still active and using nest boxes. In the Netherlands, radio tracking was undertaken between October and January in 2018 and 2019. Animals were fitted with a VHF radio-transmitter collar (in England with Type PIP3, Biotrack Ltd, Wareham, Dorset, UK and in the Netherlands with Holohil BD-2C, Holohil Systems Ltd., Ontario, Canada) and released back into the same nest box or tube immediately thereafter. Radio tracking was carried out at each site in England using Australis 26 scanning receivers and handheld Yagi three-element directional folding antennas (Titley Scientific, Coppull, Lancashire, UK) and in the Netherlands with ICOM IC-R20 or Communication Specialist Inc. R-1000 receivers and a four-element Yagi antenna (Followit Sweden AB).

#### Systematic searches

This method involves searching the woodland floor for dormouse hibernation nests by hand. This ‘fingertip search’ is normally used to locate hibernating dormice at sites where habitat is likely to be disturbed or destroyed for development purposes. This methodology often forms part of protected species mitigation licence conditions in the UK. For this study, searches were carried out through leaf litter, moss cover and ground vegetation; only moving material such as branches and stones could be done relatively easily but inspecting the surroundings of larger logs and boulders. Records collected in this way for this study were originated by the main author with additional records originating from other ecologists to increase sample size.

#### Incidental observations

These records refer to findings originating from the research team and the wider community, as well as from members of the public where hibernation sites were identified by coincidence whilst engaged in other activities (such as walking, gardening, hedge laying, woodland management) but where they were not the object of deliberate searches.

### Nest site characteristics

Once located, hibernaculum locations were recorded using handheld GPS devices (Survey 123 for ArcGIS version 1.1 for Android mobile phone in England and QFIELD version 1.2.0 for Android mobile phone in the Netherlands) with an estimated accuracy of up to 5 m and mapped using QGIS system (QGIS Development Team 2020). Using the hibernation nest as a central location, random points were then established between 10 m from the nest (to allow variations to be detected) to a maximum of 50 m radius, representing a typical dormouse home range (Bright and Morris 1991; Goodwin et al. 2018; Juškaitis 1997) using the random point generator feature in QGIS research tools. Random points were only selected within suitable dormouse habitat and features such as roads, footpaths, built-up areas and open fields (> 5 m from adjacent suitable habitat) were excluded from the modelling exercise.

At 44 hibernacula in England, leaf litter depth and distance to the nearest tree/shrub (> 2 m in height) were collected using a measuring tape whilst a visual canopy cover estimate was recorded in situ. At these locations, random points were generated in situ using Random UX for Android (UX Apps), with a set of random numbers to inform direction in degrees (1–360) and another to establish distance in metres (10–50) from nest using the same principle as the QGIS methodology described above. Additionally, temperature profiling of 19 hibernaculum locations in England and their respective random points were obtained by placing thermal data loggers (Thermochron iButton, Dallas Semiconductor, California USA) next to hibernation nests for 74 consecutive days. Temperatures were recorded hourly and from these the standard deviation, mean daily maximum, minimum, mean and variance of temperatures were calculated.

### Topography and canopy structure profiling

In England, airborne Light Detection and Ranging (LiDAR) data in the form of Digital Surface Models (DSMs) and Digital Terrain Models (DTMs) from surveys carried out in the winter months (November to April) were obtained from the UK’s Department for Environment, Food and Rural Affairs Data Services Platform (DEFRA 2020) at one-metre resolution and BlueSky International (2020) at 50 cm resolution and were resampled to 1 m resolution using average values to make the datasets comparable. For the Netherlands site, 1 m resolution LiDAR data was obtained from PDOK (a platform of geodatasets of Dutch governments, Kadaster 2021). DSMs and DTMs were then used to assess topography and vegetation structure at the landscape level surrounding hazel dormouse hibernacula and randomly selected points for comparison. At each hibernaculum location and respective random point, buffers of five metres were established using QGIS and details of microclimate were modelled for each point using the Microclima R package (Maclean et al. 2019). This package contains tools for modelling the mechanistic processes that govern fine-scale variation in temperature arising from variations in altitude, coastal influences canopy cover, cold air topographic drainage, solar radiation, surface albedo and wind speed.

**Fig. 2:**
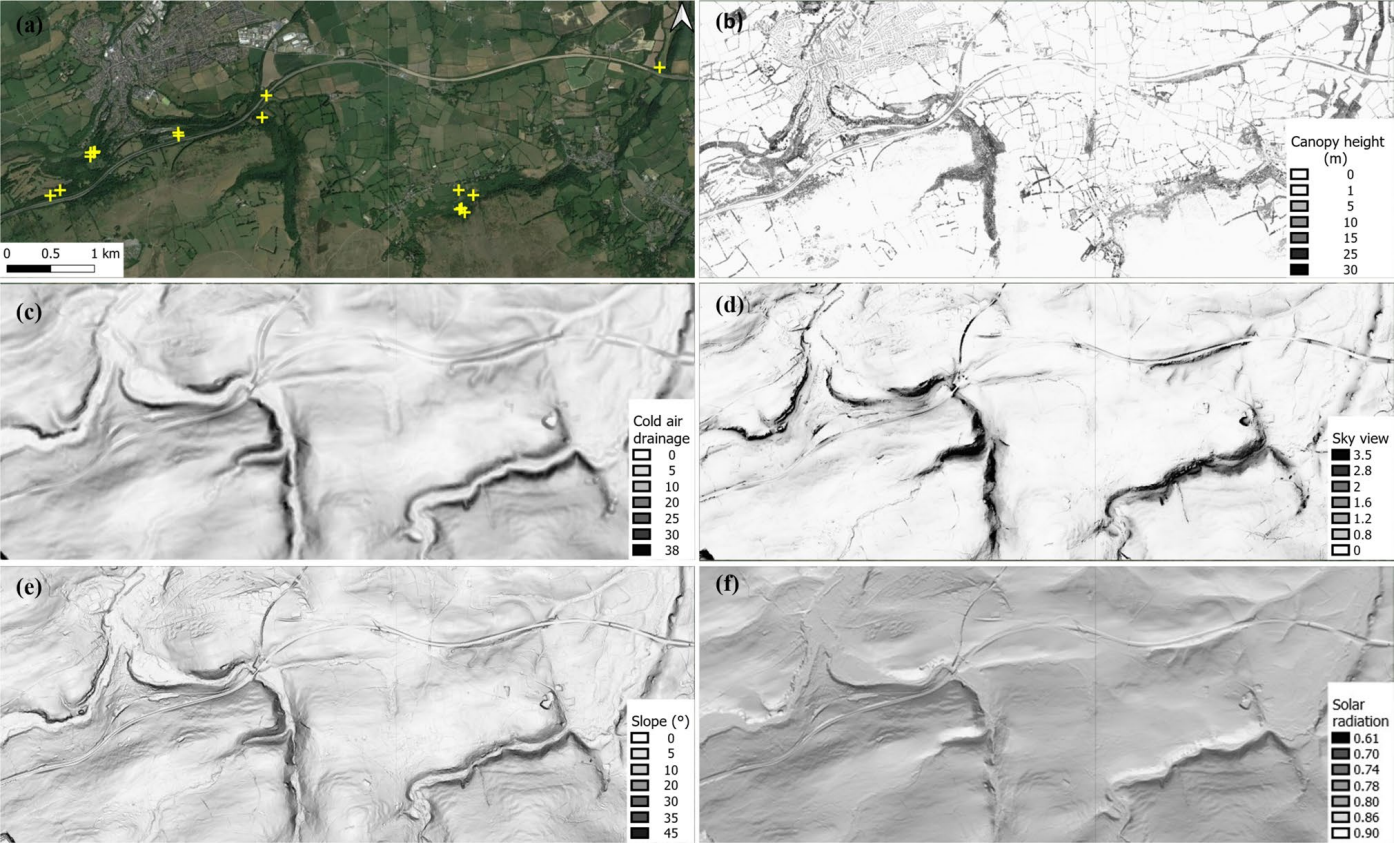
Maps of an area of southwestern England against backgrounds of (**a**) satellite imagery (Google Maps 2021) showing hibernaculum locations marked with yellow crosses, and spatial variation in **b** canopy height, **c** cold air drainage, **d** sky view, **e** slope and **f** solar radiation

The information extracted from the LiDAR data and Microclima package (Fig. 2) is defined as follows:

#### Canopy height and cover

Forestry structure is known to be an important factor in the habitat of the hazel dormouse (Goodwin et al. 2018). Canopy height and cover were obtained by subtracting DTMs from DSMs using “raster” and “rgdal” packages in R (R Core Team 2017). Canopy height is the value of difference between the DSM and DTM; canopy height was calculated at the focal pixel and minimum, mean and maximum values were calculated from within a 5 m radius of the focal pixel. Canopy cover is defined here as the proportion of pixels with a canopy height above 0.1 m within a 5 m radius of the focal pixel. These canopy metrics will also reflect, to some extent, variation in the amount of light penetrating the canopy. Buildings were removed from the canopy height and cover calculations by using a buildings’ raster (as detailed in Ordinance Survey 2022) downloaded from Digimap (2021) under an educational licence as a masking layer.

#### Slope

Field observations suggested that dormice selected sites well drained, on a slope and not prone to flooding. It refers to the steepness or the degree of incline of a surface and calculated using DTMs at 1 m resolution. Slope is also associated with drainage and hydrology and was extracted using the terrain function in the “raster” package.

#### Sky view

Linked to topography, sky view is the proportion of sky dome obscured by terrain from the sampled location excluding canopy and is calculated using DTMs at 1 m resolution with the Microclima package using the skyviewtopo function. Sky view can be a proxy for landscape position and for radiation exchange with the atmosphere and radiative warming of the ground surface, which can be relevant for hazel dormice when seeking a cool hibernation site with stable temperatures. Extracted values were adjusted to facilitate illustration using the following formula: 1-sky view × 10^5^ resulting in higher values in deep valleys and lower on flat ground or hill tops.

#### Solar radiation

Considered to be an important factor for hibernaculum selection as existing literature suggests that dormice require low constant temperatures to hibernate. This index was calculated using the solarindex function in the Microclima package that measures the proportion of direct beam solar radiation that, as described by Bennie et al. (2008), is the main component of ground surface energy balance and influences ecologically critical factors of microclimate, including near-surface temperatures, evaporative demand and soil moisture content. Solar radiation indexes were calculated based on the position of the sun integrated across daylight hours on 21st December.

#### Cold air drainage potential

Is the potential for air in contact with terrain surfaces to be cooled and to flow downslope and/or downvalley and, in the context of this study, may also be a proxy for both cold air (temperature), soil moisture and hydrological drainage. Cold air drainage potential is used in this context to indicate both aggregate downslope (katabatic) and downvalley flows and was calculated using the Microclima package using the pcad function. It can be a particularly important element for ground-level hibernators because of its potential to impact ground and ambient temperature as well as moisture and hydrological drainage.

Once the values of the remotely sensed terrain and canopy variables were obtained, the intercept and coefficients for each variable from the top model (lowest AIC value) for each country were used in the Raster Calculator function in QGIS to calculate an index of the relative probability of a nest in each map pixel. Vegetation cover (> 0.1 m in height) was used as a masking layer, restricting modelled potential sites to areas with canopy cover. The output layer illustrates predicted suitable hibernation locations with topographic, microclimate and vegetation characteristics matching those of actual hibernacula.

### Data analysis

Data collected in situ: Wilcoxon tests were used to test for differences between recorded variables at each paired hibernacula locations and random points on the distance to the nearest tree, temperature and leaf litter depth between hibernacula and random points as well as estimated canopy cover collected in situ and remotely sensed.

Remotely sensed variables: To test whether hibernaculum locations differed significantly in site characteristics from the paired random locations a generalised linear mixed model was used to investigate the status of each location (hibernaculum or randomised) as a function of the remotely sensed variables. Remote sensed variables were rescaled to a mean of zero and unit SD and were included in the analysis as fixed factors. A site location code (shared between each pair of hibernaculum location and random point) was included as a random effect to account for the paired experimental design. General linear mixed models were firstly run separately for both England (*n* = 62), and the Netherlands (*n* = 21) and then combining both datasets, using pooled hibernation sites and random points with all possible combinations of remotely sensed variables (slope, canopy height and cover, cold air drainage). Best models selection followed using R Package ‘MuMIn’ (Bartón and Barton 2020) and a model averaging procedure (Johnson and Omland 2004) to identify key explanatory variables. A “best” set of all models with AIC (Akaike’s Information Criterion) values within two units of the lowest AIC value were selected, and effect sizes were calculated from this model set as the Akaike-weighted average slopes of the standardized variables across the top model set. The relative importance of explanatory variables was calculated as the proportion of the “best” set of models containing each explanatory variable.

## Results

**Fig. 3:**
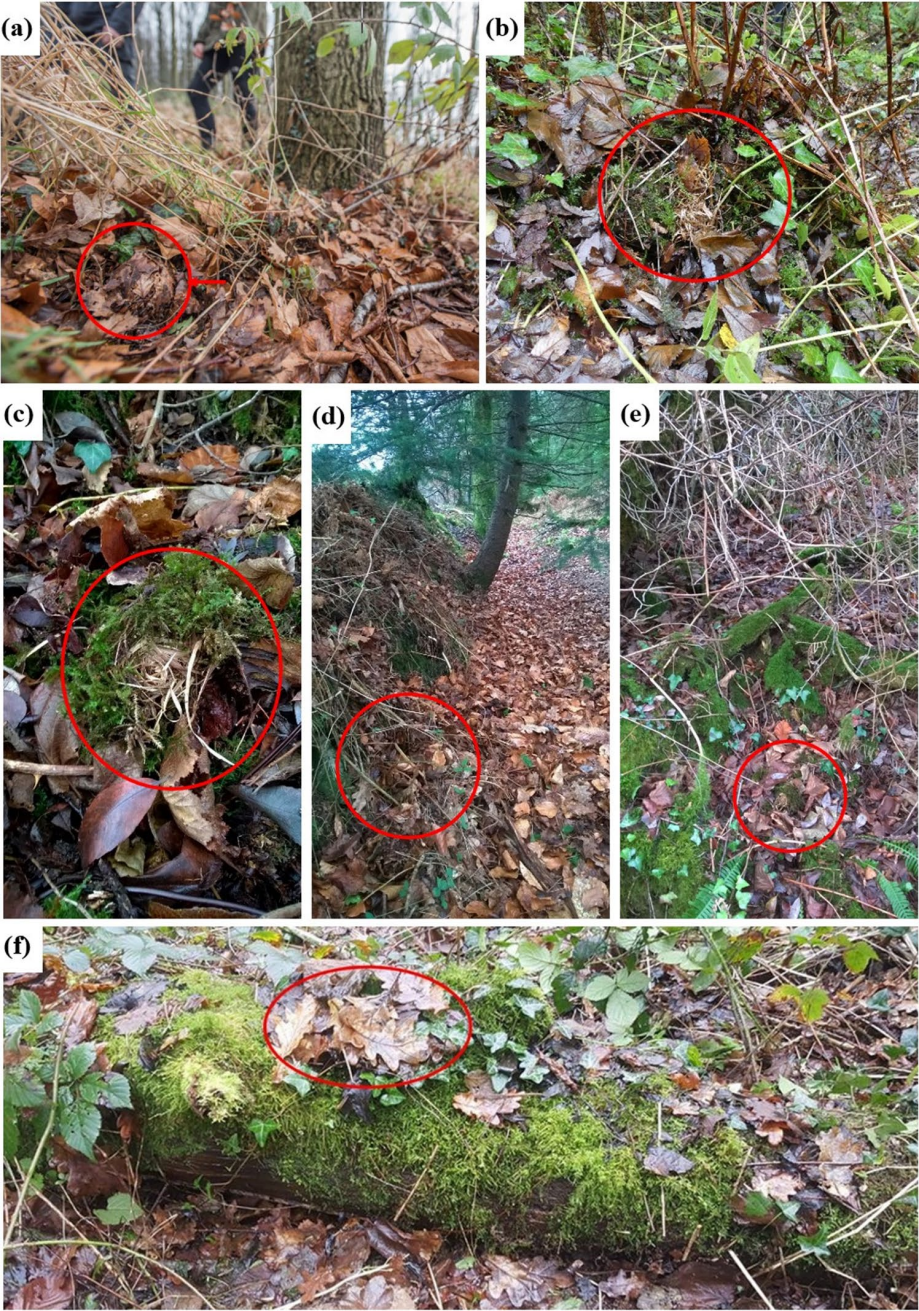
Examples of dormouse hibernacula in different locations: **a** Nest in leaf litter in the Netherlands, **b** nest above ground anchored at the base of a fern in waterlogged ground, **c** conspicuous nest in leaf litter, **d** hibernaculum built on hedge bank 30 cm off the ground on flood-prone area, **e** hibernaculum sited on concrete wing wall adjacent to highway and, **f** hibernaculum constructed on top of branch on flat ground

Hazel dormice hibernacula were predominantly located among leaf litter on the woodland floor, with varying degrees of concealment (Fig. 3). In England, dormice largely hibernated in the leaf litter on the woodland floor, occasionally under ferns and patches with dead bracken fronds, often metres away from the nearest tree or shrub. In hedgerow habitats, we found that dormice were often nesting nearby on adjacent grassland or field edges as well as within the hedge itself, at the base of hazel stools. Similar results were obtained in the Netherlands. There, most hibernacula were found in the leaf litter and covered with leaves on the sparsely vegetated woodland floor. Three nests were found in a hedgerow, also adjacent to field edges.

At sites with low gradients or waterlogged ground, some hibernation nests were built higher off the ground, on vegetation (e.g. ferns and grass tussocks) or other physical features (e.g. hedge bank and fallen branches), to reduce exposure to excessive damp or being flooded. We recorded a small proportion of the hibernacula (*n* = 6) constructed in small depressions dug out in the ground in the form of a ‘cup’ to support the bottom half/third of the nest. Some (*n* = 4) in leaf litter on sloped ground securely positioned in the root systems of trees where the soil has eroded underneath. It was also noted that dormouse hibernacula in woodland seemed to have been selectively avoiding areas with clear evidence of other small mammals, particularly bank voles *Myodes glareolus*, such as feeding stations and network of tunnels under moss and/or leaf litter.

### Data collected in situ

Observed hibernaculum locations at UK sites did not differ from random points in distance to the nearest tree or leaf litter depth (all *p* > 0.1). There were also no differences in canopy cover at hibernaculum locations and random points, whether the cover was estimated remotely or in situ. However, the average daily mean temperature at hibernaculum locations (*n* = 19) was slightly lower (0.5 °C) than at random points (*p* = 0.038), but there were no significant differences in minimum, maximum, or daily variance in temperature (all *p* > 0.1).

### Remote sensing

**Fig. 4:**
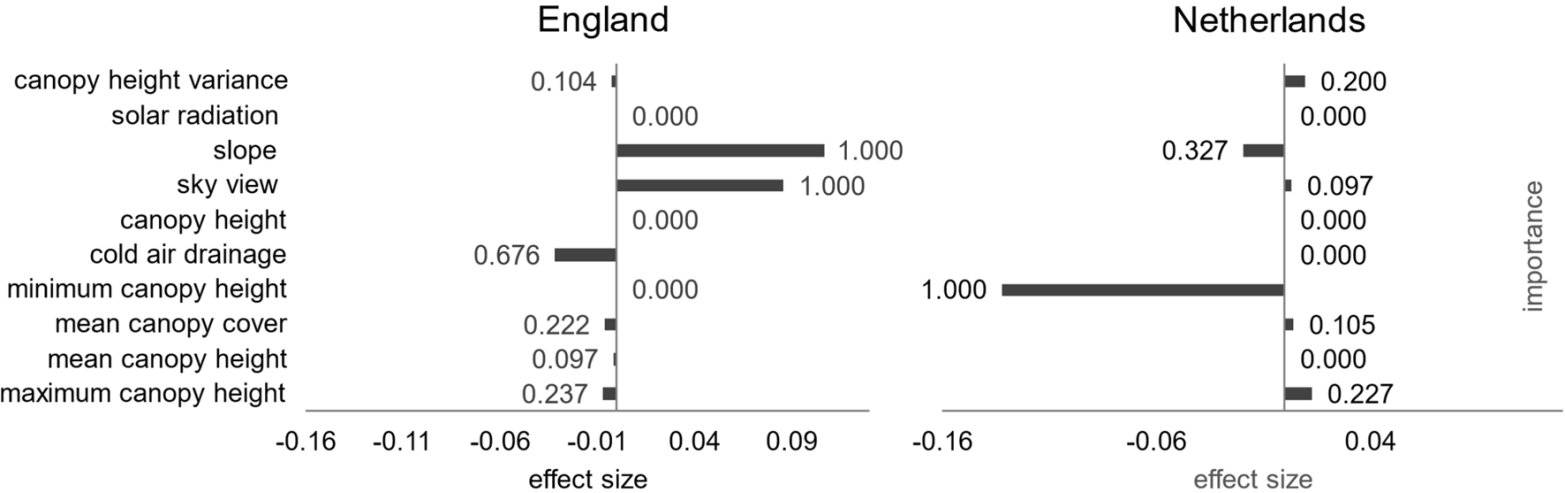
Summary of factors affecting the location of hazel dormouse hibernacula in England and the Netherlands. Effect sizes are from the top model set analysing variation in standardized variables from observed hibernaculum locations and random points nearby. The bars represent the effect size, calculated as the magnitude and direction of the Akaike-weighted coefficients of terms in the set of models within 2 AIC units of the best model. Figures at the end of bars represent the relative importance, defined as the proportion of models within the set in which the term is included. Variables with zero weight and zero effect were tested but did not feature with the top model set in one or both locations

Environmental characteristics surrounding hibernacula differed markedly between the two countries (Fig. 4). In England (*n* = 62), hibernaculum locations were positively related to slope gradient and sky view. Here, dormouse hibernacula were found predominantly on sloping terrain, avoiding deep valleys (higher sky view values and slope gradient). When analysing individual search methods utilised in England (telemetry. systematic search and incidental finds), the null model featured as the top model.

In the Netherlands (*n* = 21), both slope and sky view had low importance, but the negative relationship with the percentage of minimum canopy cover within a 5 m radius of the hibernacula indicates that there is an effect of canopy cover on nest sites that is not evident in England (Figs. 3, 4), suggesting that animals preferred locations by the woodland edge or near gaps in the canopy.

**Fig. 5:**
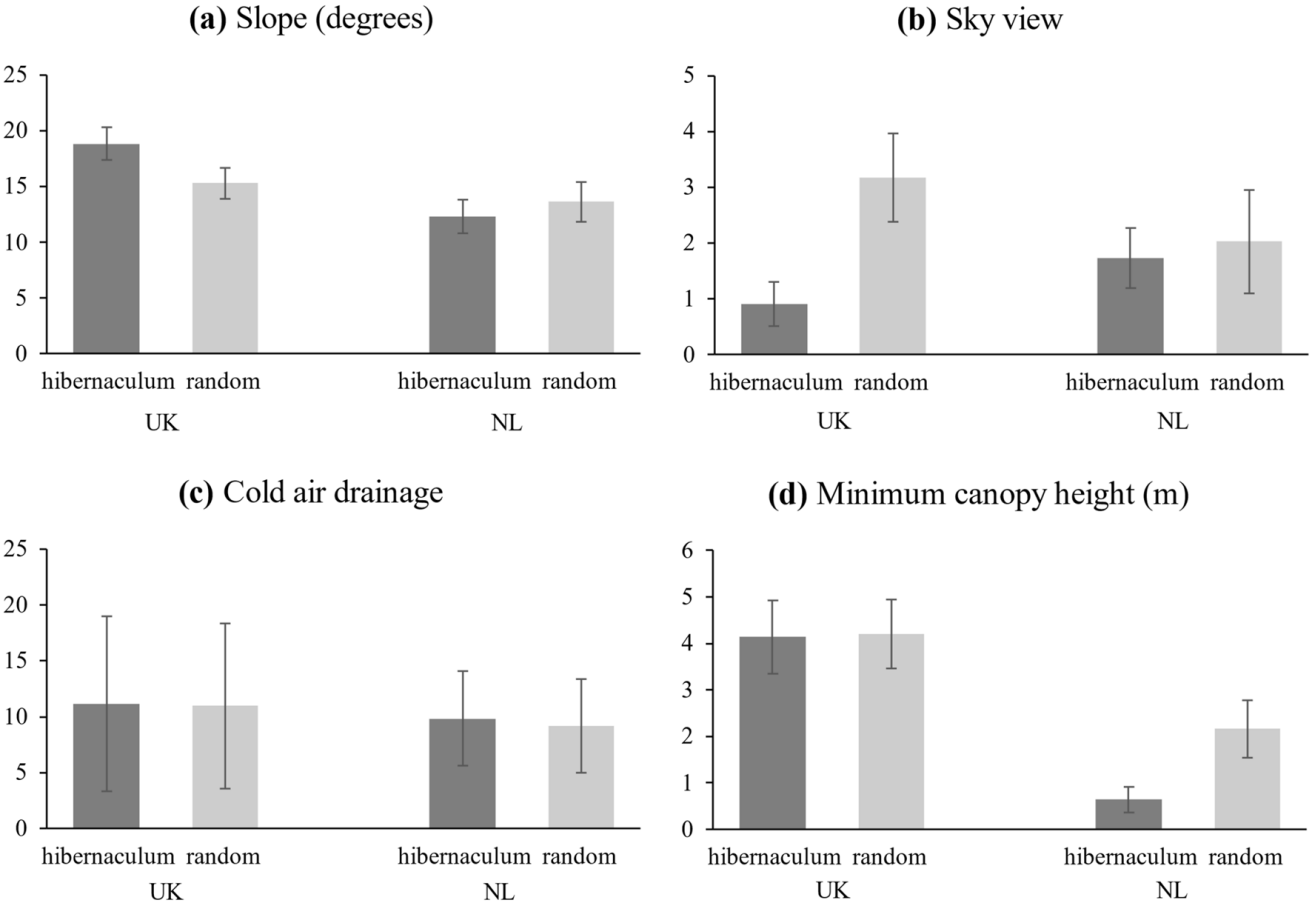
Summary of variation in remotely sensed variables measured at hibernation site locations and associated random points in England: **a** slope, **b** transformed sky view and **c** cold air drainage, and the Netherlands: **d** minimum canopy height. Columns indicate the mean and error bars represent the standard error

When both countries were analysed separately, the null models were outside the top model set, indicating that remote sensed variables had important, but contrasting, effects in both England and Netherlands (Fig. 5). However, with the combined dataset, and interaction terms for the country, we found that the null model (including no spatial variables) was within the top model set (selected using AIC values). Therefore, consistent patterns in nest location across both countries could not be detected with confidence.

**Fig. 6:**
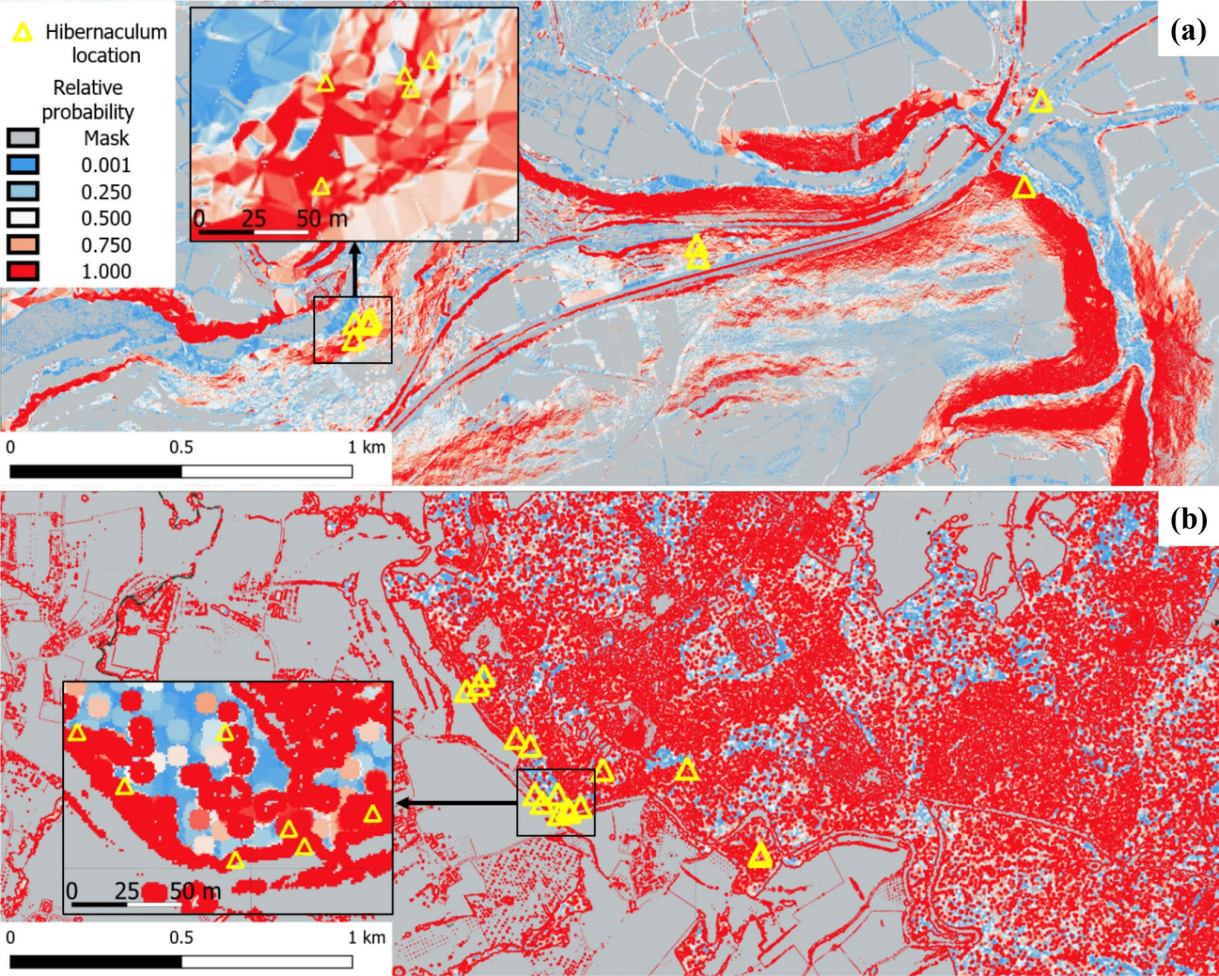
Example of predictive models of the suitability for hazel dormouse hibernaculum across landscapes in England and the Netherlands with inset maps. Observed hibernacula are marked by a yellow triangle for illustration in areas in **a** England—based on sky view and slope, and **b** Netherlands—based on minimum canopy height, highlighting the relative probability of the area to match habitat characteristics according to the relevant remotely sensed variable(s) of nearby hibernaculum locations

Based on the remotely sensed predictive variable values, environmental conditions models based on the relative probability of finding dormouse hibernacula were created, indicating areas of similar characteristics across the landscape (Fig. 6).

## Discussion

One of the great advantages of using LiDAR in ecological models is its ability to characterise a three-dimensional habitat structure of terrestrial environments in fine detail across large areas (Vierling et al. 2008). Despite the limitations that may affect the accuracy of micro-topography and crown characteristics on tree height estimations of LiDAR canopy height models (Alexander et al. 2018) our remote sensed data was found to be effective in identifying topographic and vegetation features favoured by dormice in selecting hibernacula sites. We were able to demonstrate how specific features used by the dormice when selecting hibernaculum location differed in distinct landscapes. In addiiton, that there is an apparent difference in the variables that have explanatory power between the two studied countries and, although not readily transferable, they are able to indicate ecological variations at different scales. Additionally, our results indicated that remote sensing proved more efficient at identifying highly localised environmental characteristics in the wider landscape that may be important for hibernaculum selection when compared to habitat data collected in situ alone.

Similar to results reported by Cartledge et al. (2021) where slope featured as an important habitat descriptor for dormice during the active season, we found that topography was a key factor in hibernaculum selection in England with both slope and sky view variables distinctly featuring in top model sets. There, dormice built their hibernacula on slopes, avoiding narrow bottoms of deep valleys and largely building their hibernacula in leaf litter on the woodland floor. Additional subjective observations in the field suggest that where hibernacula were recorded on flat/low gradient landscapes, prone to or with the potential of being waterlogged, dormice built their nests slightly higher on grass tussocks, hazel stools, ferns or on top of fallen branches to avoid excessive dampness or being flooded during the hibernation period. In the Netherlands, although slope derived from the DTM did not prove a significant predictor of hibernacula location in this study, in a previous analysis using in situ field data gathered on the same nest-site dataset, Lemmers et al. (2022) found a significant difference in slope between nest sites and random locations, consistent with our observations in the English sites. This suggests that the field observations may have detected sloping features that were not well represented in the LiDAR data. Their study used a similar method but selected random locations within a 20 m radius, rather than the 50 m used in this study.

Cold air drainage, which can also be a proxy for water drainage, had a negative effect in England, suggesting dormice preferred to locate their hibernacula away from cold air flow and avoided areas such as ditches, trenches and gullies that may be a water conduit/accumulator. The positive effect of the sky view variable exerts, also indicate that locations such as the bottoms of valleys and gorges in general, where often cold air drainage values are higher (Maclean et al. 2019), were avoided. In the Netherlands, despite sloped terrain being present in the landscape, neither slope nor sky view index had explanatory power perhaps associated with the fact that England is subject to higher annual rainfall.

Surprisingly, solar radiation did not feature as a relevant variable capable of influencing hibernaculum location in neither country. Indeed, in England, some hibernacula were located under closed canopy away from forest edges, whilst others were fully exposed to sunlight, often recorded away from woody vegetation cover on open ground. The fact that solar radiation had little importance or effect fact is reflected by the negligible effect canopy cover had on hibernaculum location in both countries. Perhaps, due to nests being well insulated and constructed under leaf litter and other vegetation (Gubert et al. 2022) as well as the seasonal effect, solar radiation had no effect on hibernating animals during low temperature spells. With regards to canopy cover, many of the hibernaculum locations in England and the Netherlands share similar characteristics as described by Verbeylen et al. (2017) in Belgium, where they were often found in open areas within or surrounding woodland blocks. Dormice also seemed to avoid localised areas of woody scrub cover such as bramble *Rubus fruticosus*, mammal burrows and log piles, possibly as a measure to prevent predation or disturbance by other animals that actively seek out these features for shelter.

Whilst canopy height (vegetation above > 0.1 m) was not an important factor for hibernaculum location in England, dormice seem to be clearly favouring woodland edges in the Netherlands. The effect size and importance of minimum canopy height extracted from the top model set showed a pronounced difference between the two countries. However, it is important to note that in the Netherlands dormice were captured and radio-collared in nest boxes placed along woodland edges but even when considering their relatively small home ranges, the animals still opted to hibernate nearer to the woodland edge than randomised points in the vicinity of their capture locations. Similar observations were made with ground squirrel in North America by Goldberg et al. (2020) as hibernaculum locations had more canopy cover than random points but had less cover than sites used during the active season.

We have also found that data collected in situ on leaf litter depth, distance to nearest trees and canopy cover did not have significant predictive power in identifying hibernaculum locations. These are habitat features mentioned in the literature (see Bright et al. 2006) that were considered important for hibernating dormice but do not alone seem to distinguish hibernaculum locations. Whilst some of our hibernacula shared similarities with the existing literature by being found at locations such as coppice stools, under moss and leaf litter, we did not find any under large stones, boulders, logs, tree roots or animal burrows. We found that the distance between hibernaculum and the nearest tree trunk or vine were not significant and mean distance was nearly three times greater (*n* = 44) than the 0.5 m reported by Bright (1992). Also, we were able to establish that the accuracy of the canopy cover of the remotely sensed data could be compared with the information collected in situ.

Measured temperature had a minor influence on hibernaculum location. The average daily mean temperature at hibernaculum locations (*n* = 19) was slightly lower than at random points collected at the hibernacula, indicating that dormice chose to hibernate at sites with lower average mean temperatures. This finding corroborates Bright and Morris (1996) suggesting that dormice seek out a cool place on the ground to hibernate.

Our results suggest that using remotely sensed data can be useful in detecting localised topographic features and climatic conditions that may be relevant to species that favour specific conditions during their life cycle or at certain life stages. With the relatively simple method and freely available high-resolution data, this methodology has the potential to provide more accurate species distribution and habitat suitability models particularly if applied to a larger sample size across the species range.

As highlighted by Trout et al. (2012), hibernating dormice are vulnerable to disturbance during hibernation at ground level, especially during forestry operations. The predictive models can be useful to inform woodland and habitat management as well as development projects, potentially focusing survey efforts based on relevant habitat characteristics.

We found that the extent to which different remote sensed variables have explanatory power varied between countries and therefore cannot be considered species-deterministic but context-dependent. The explanatory variables were able to predict suitable dormouse hibernaculum locations across large areas of the landscape indicating that these can be quite abundant and also that dormice may be flexible or tolerant with regards to specific topographic features and environmental conditions in which to hibernate. This flexibility may also be an effective strategy to avoid predators such as birds, badgers, foxes that could be biased to look for their hibernaculum at specific places on the woodland floor (e.g. next to tree trunk, on specific gradients or under closed canopy) whilst reiterating the benefit that a mosaic of microhabitats is capable of offering suitable conditions for hibernation.

Since the hazel dormouse and its habitat are fully protected throughout its European range, the modelling approach demonstrated here may prove useful in situations when the habitat is being disturbed or destroyed by, for example, development, habitat management and forestry activities. In England and Wales, derogation licences issued to ensure legislation compliance against intruding activities within dormouse territory in the winter often require that systematic searched are undertaken to identify and/or move dormouse hibernacula to ensure animal welfare. The predictive models presented in this study could highlight areas where searches should be focused, especially for large sites.

Given the diversity of locations in which hibernating dormice were found, it is likely that suitable conditions for hibernation is relatively abundant within their home range as illustrated in our predictive models. Bright and Morris (1996) suggested that suitable places for hibernaculum are available in most woodlands and are unlikely to be a limiting factor, and their findings also highlight that during this period animals might then be vulnerable to floods, trampling and predation.

The results of this study demonstrated for the first time that there are specific variables favoured by dormice when choosing hibernaculum locations. Whilst conservation efforts such as habitat creation, enhancement and management are of great importance for dormice, they have the potential to negatively impact local populations when carried out indiscriminately during the hibernation season. The use of predictive models to highlight areas where dormice are likely to site their hibernacula can be a useful tool to inform and guide woodland management as well as large-scale forestry operations to safeguard local populations.

## Data Availability

The data that support the findings of this study are available from the corresponding author, LG, upon reasonable request.
